# Interventricular Septal Hematoma Complicating Left Bundle Branch Area Pacing: A Case Report—The Devil Is Not So Black as He Is Painted

**DOI:** 10.3390/jcdd11020052

**Published:** 2024-02-01

**Authors:** Paolo Pastori, Fabrizio De Rosa, Francesco Vitali, Andrea Fasulo, Giovanni Tortorella, Monica Pastore, Michele Malagù, Matteo Bertini

**Affiliations:** 1 Cardiology Unit, Ospedale di Fidenza, AUSL di Parma, 43036 Fidenza, Italy; 2 Cardiology Unit, Sant’Anna University Hospital, Department of Translational Medicine University of Ferrara, Via Aldo Moro 8, 44124 Ferrara, Italy

**Keywords:** left bundle branch area pacing, interventricular septal hematoma, ventricular arrhythmias, echocardiography, case report

## Abstract

Background: This case report outlines the presentation of an emerging complication arising from left bundle branch area pacing (LBBAP). Case summary: A 43-year-old male with no history of cardiac problems experienced recurrent episodes of syncope with no prodromal symptoms. During monitoring in the emergency department, the patient underwent an episode of asystole, leading to LBBAP implantation. The procedure encountered technical challenges, resulting in an interventricular septal hematoma and subsequent ventricular arrhythmias. Despite initial concerns, conservative management led to resolution, demonstrated through echocardiographic follow-ups. Discussion: This report underscores the significance of ventricular arrhythmias as indicators of interventricular septal hematoma, providing insights into its diagnosis, management, and implications for LBBAP procedures.

## 1. Introduction

In recent years, left bundle branch area pacing (LBBAP) has emerged as a novel and more physiologically advantageous pacing technique compared to traditional right ventricular pacing and His bundle pacing (HBP). LBBAP has arisen as a compelling alternative, overcoming numerous limitations associated with HBP in terms of feasibility, electrical parameters, and device settings. LBBAP involves using a ventricular transseptal route for lead placement and is used for both bradyarrhythmia and heart failure indications. Directly pacing the left bundle allows for a more physiologically natural activation sequence of the ventricles, aiming to mitigate the ventricular dyssynchrony often encountered with traditional pacing methods. Even patients with preserved left ventricular ejection fraction who are going to experience slightly high percentage of ventricular pacing will probably benefit from conduction system pacing due to better biventricular efficiency during LBBAP. Notably, the rate of complications associated with LBBAP is comparable to that reported for biventricular cardiac resynchronization therapy implantations [1]. However, in the MELOS study, a multicenter European registry-based observational study involving 2533 patients across 14 high-volume European centers, interventricular septal hematoma was not reported as a complication [1] and the overall incidence of acute and late complications was 11.7%. Specific complications related to the ventricular transseptal route of lead placement were observed in 8.3% of patients, predominantly attributed to intraprocedural perforation into the left ventricular (LV) cavity. Unlike traditional RV pacing or HBP, recent case reports [2,3,4] have described interventricular septal hematoma as a novel and rare complication of LBBAP. This complication, however, was not observed in the MELOS study [1]. The septal perforating arteries are vessels that usually originate from the anterior and inferior interventricular arteries. In their initial segment, these vessels are larger, but they gradually become thinner in the middle of the interventricular septum which is the common target area for LBBAP. In certain cases where electrical parameters are not acceptable, or the interventricular septal anatomy does not allow lead placement in the middle part of the interventricular septum, a more anterior or posterior lead position may be attempted. However, this poses the risk of engaging perforating septal branches, particularly where they have a larger caliber. The clinical presentation of interventricular septal hematoma, typically, involves persistent chest pain, sometimes accompanied by ECG changes, dyspnea, and elevated troponin levels, exceeding those typically observed following routine LBBAP procedures. In one case, a significant pericardial effusion was observed [3], while another case presented with near-complete obliteration of the right ventricle (RV) [4]. All reported cases were associated with the use of the traditional lumenless pacing lead. Conservative management strategies were employed in two out of three reported cases, leading to complete resolution within 4–6 weeks. The development of interventricular septal hematoma is believed to be caused by injury to a small branch of a perforating septal artery during lead penetration into the interventricular septum. The transseptal route of the LBBAP lead exposes it to potential damage of vascular structures, leading to this new procedural complication [5].

## 2. Detailed Case Description

**Figure 1:**
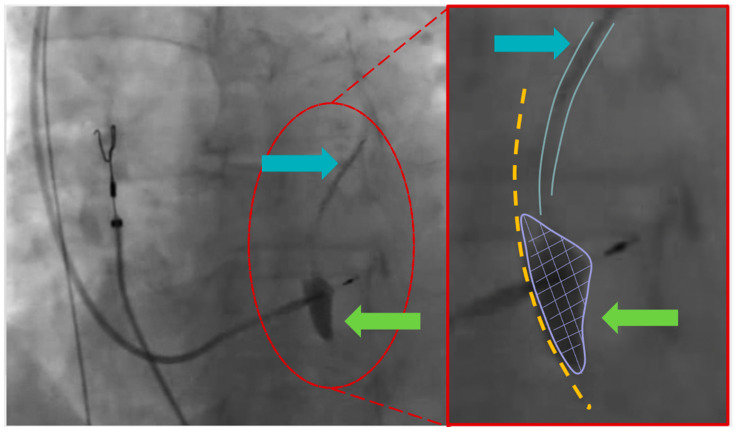
**Left** part (left anterior oblique view—35°): The image displays the second positioning attempt of the ventricular lead. The green arrow indicates the observed stagnation of contrast inside the septum. The light blue arrow highlights the septal branch. **Right** part (enlarged image): The right part of the interventricular septum is marked with a yellow dot line. The green arrow and purple line emphasize the stagnation of contrast directly linked to the septal branch (depicted by the light blue lines and arrow).

**Figure 2:**
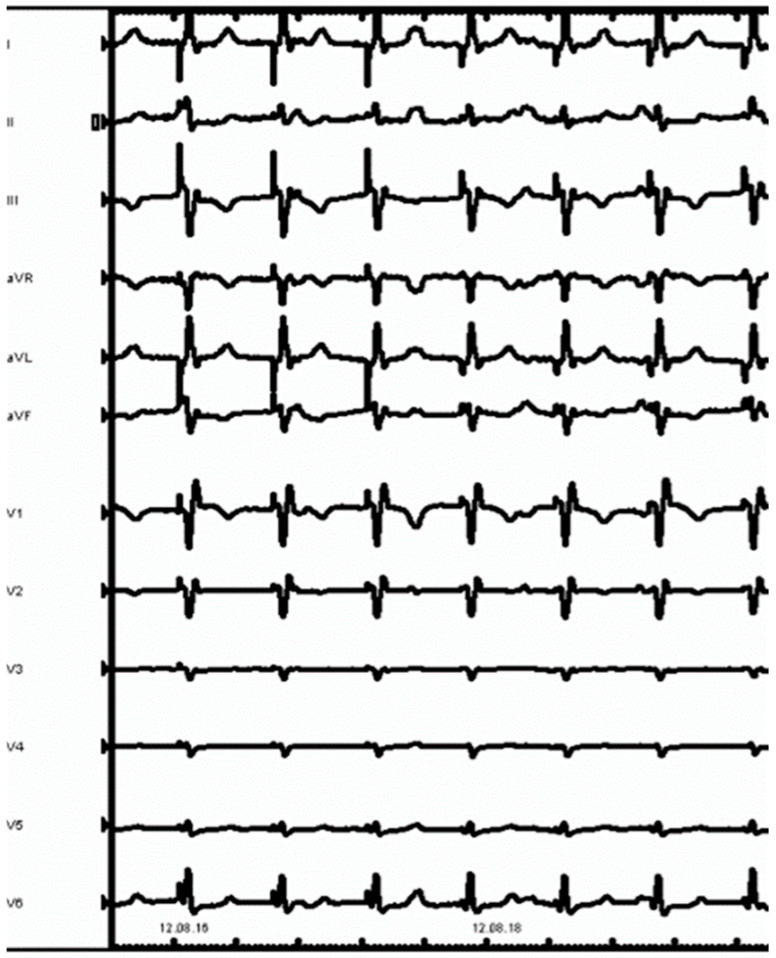
ECG obtained after the final lead placement showed a paced QRS with LBBPA morphology.

**Figure 3:**
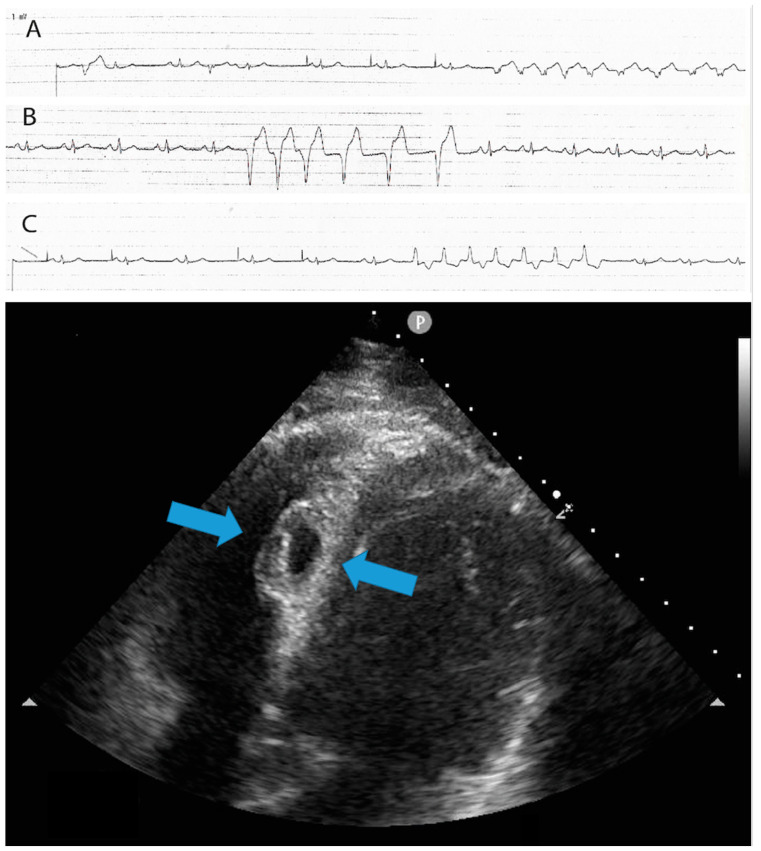
The upper part of the image shows the presence of non-sustained ventricular tachycardia (NSVT) and ventricular ectopic beats of different morphologies, which developed at the end of the procedure and persisted the following day. The lower part of the image displays a 4-chamber view echocardiogram conducted the day after the procedure. It reveals the presence of an interventricular septal hematoma measuring 17 × 10 mm, which protruded into the right ventricle (RV)—light blue arrows. A, B and C strips shows non-sustained ventricular tachycardia with different morphologies.

**Figure 4:**
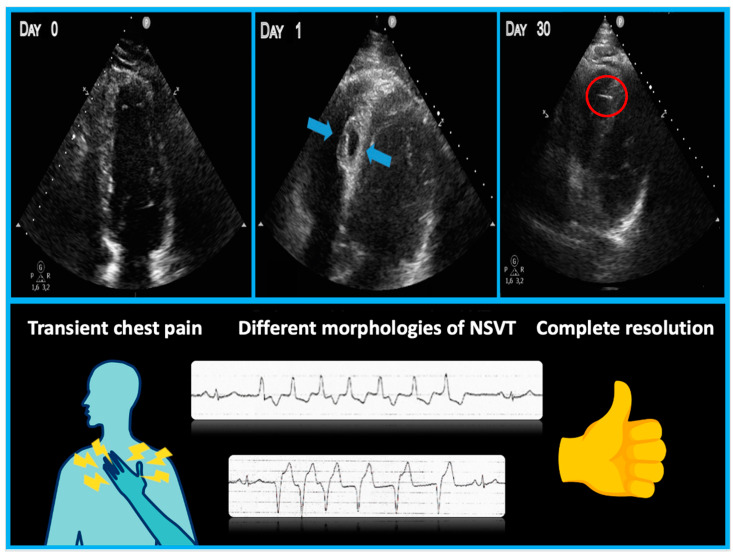
Summary figure (graphical abstract). The **upper** part of the image depicts the evolution of the septal hematoma (light blue arrows) in the 30 days following the implant procedure until complete resolution. The **lower** part shows the associated symptoms and ventricular arrhythmias observed during the follow-up period.

A 43-year-old male with no prior history of cardiac issues was referred to our hospital after experiencing two recent episodes of syncope with resultant trauma, without any warning signs. The patient had also reported two previous episodes of syncope without prodromal symptoms several months ago. The patient did not take any medications before hospitalization and had an uncle who underwent pacemaker implantation for syncope at the age of 74. The initial electrocardiogram (ECG) revealed sinus bradycardia with a heart rate of 46 beats per minute and normal atrioventricular conduction. Additionally, an echocardiogram was performed, ruling out any structural myocardial diseases. While undergoing ECG monitoring in the emergency department, the patient experienced another syncopal episode, during which a sinus arrest episode with 18 s period of asystole was documented. As per our standard practice, we screened for major causes of sinus arrest, including sleep apnea syndrome, infection, electrolyte alterations, and a familial history of sudden death or pacemaker implantation at a young age and the screenings were all negative. Unfortunately, the patient did not undergo genetic analysis. The day after the syncopal episode, the patient underwent left bundle branch area pacing (LBBAP). The pacing indication was of prolonged sinsus arrest, nevertheless, the decision to perform physiological pacing was taken, despite the possibility of programming managed ventricular pacing algorithms due to the young age of the patient and the possibility of high ventricular pacing being needed in the future. During the implantation procedure, a first attempt was made in the basal interventricular septum to perform LBBAP with a lumenless pacing lead (Medtronic 3830) but the electrical parameters were unsatisfactory. On the first attempt, we were unable to achieve left bundle capture due to the suboptimal progress of the lead through the septum. Subsequent additional rotations resulted in a sudden decrease in unipolar and bipolar sensing and an increase in threshold. Therefore, a second attempt was made, and the lumenless pacing lead was successfully screwed into the mid-basal interventricular septum, achieving optimal ECG parameters consistent with LBB pacing (right bundle branch morphology, V6 R-wave peak time 61 ms, V6–V1 interpeak interval 41 ms, and QRS duration 123 ms). The patient experienced chest pain during this time. As it is not our usual practice, we performed the contrast injection after lead placement because the patient experienced chest pain. However, during the contrast infusion, stagnation of contrast was observed inside the septum, and a rupture of a septal branch was also visualized (Figure 1). The ECG did not reveal any ST changes, and the patient remained hemodynamically stable. The pacing lead was then repositioned more distally towards the mid-apical septum, with electrical parameters that were less optimal but still acceptable (right bundle branch morphology, V6 R-wave peak time 72 ms, V6–V1 interpeak 32 ms, and QRS duration 132 ms) (Figure 2). Gradually, the chest pain subsided within a few minutes and completely resolved within half an hour. An immediate post-procedure echocardiogram revealed the pacing lead’s proper position with no abnormalities or pericardial effusion. However, non-sustained ventricular tachycardia (NSVT) and ventricular ectopic beats of different morphologies developed at the end of the procedure and persisted the following day (Figure 3, Panel A). A bedside echocardiogram conducted the day after the procedure ruled out pericardial effusion but identified an interventricular septal hematoma measuring 17 × 10 mm that protruded into the right ventricle (RV). This hematoma remained unchanged in a subsequent echocardiogram performed two days later (Figure 3, Panel B). The troponin levels at admission were negative. After the procedure, we observed a slight increase in TnI-Hs levels with a peak of 131 ng/mL. After a multidisciplinary discussion, a conservative management approach was chosen due to the patient’s complete absence of symptoms and stable hemodynamics. Given the patient’s stable condition, coronary angiography was not deemed necessary. The initiation of antiarrhythmic treatment with sotalol resulted in the complete regression of NSVT and ventricular ectopic beats. Subsequently, the patient was discharged three days after the pacemaker implantation. During the routine one-month follow-up visit, the pacemaker interrogation revealed no ventricular arrhythmias and the electrical parameters remained stable and optimal. The echocardiogram confirmed the complete resolution of the interventricular septal hematoma (Figure 4). As a result, sotalol was discontinued. After a three-month follow-up, the patient remained completely asymptomatic, with no further ventricular arrhythmias, and the electrical parameters of the pacemaker remained stable and optimal, as observed during the time of implantation.

## 3. Discussion

Conduction system pacing, inclusive of HBP and LBBAP, constitutes a physiologically driven pacing modality that initiates myocardial stimulation through specialized conduction fascicles. Its primary objective is to avoid the electrical and mechanical dyssynchrony associated with traditional RV apical pacing and its consequential adverse effects. Despite the widespread adoption of LBBAP, there remains limited understanding of its complications. The primary complications include septal perforation or lead fracture, while a less-explored complication involves septal hematoma related to damage to the septal arteries, posing challenges in its diagnosis and management. In the present case, for the first time, ventricular arrhythmias were described as a clinical hallmark of interventricular septal hematoma, indicating possible myocardial injury resulting from irritation or stretching caused by a large hematoma within the relatively thin interventricular septum. The ventricular ectopic beats or NSVT in the present case differed from the typical RBBB morphology, indicating LBB irritation. Moreover, different morphologies of ventricular ectopic beats and NSVT were observed, possibly due to irritation of a large part of the interventricular septum. Despite the concerning clinical presentation, conservative management was employed in the present case, in line with the recommendation of Trivedi et al. Echocardiographic follow-up was crucial and sufficient for diagnosis, assessment of hematoma size, and monitoring of its resolution. In more severe cases with pericardial effusion, persistent chest pain, or the need for coil embolization of a ruptured arterial branch, computed tomography angiography or coronary angiography may be warranted. To prevent interventricular septal hematoma, it is recommended to avoid the antero- and postero-septal route. The lumenless leads used in this particular case, due to the isodiametric helix, were easier to place deep in the interventricular septum than the stylet-driven leads. All the reported cases of septal hematoma involved this kind of lead. Despite this, both types of leads need to be placed deep inside the IV septum to perform LBBP correctly. Therefore, the risk of engagement with septal perforating arteries is probably the same but we also have to consider that stylet-driven leads have greater diameters, so the risk of collateral damage to septal structures, such as septal arteries, could be slightly more elevated. An additional tip to minimize the risk is to ensure that lead II, after confirming a QRS complex more positive than lead III, does not show a completely positive or completely negative QRS pattern but, instead, a small negative or positive component should be present. The complete negativity of the QRS complex in inferior leads suggests the pacing origin from the inferior part of the septum, whereas complete positivity in inferior leads indicates an origin from the anterior part of the septum. Positive/negative QRS complex in the inferior leads suggests the origin of the pace mapping for the mid-septum. Employing this paced QRS characterization allows for the avoidance of excessively anterior or posterior lead placement, mitigating the risk of interference with the larger septal perforating arteries.

## 4. Key Take-Home Messages:

- Minimize the number of attempts during lead positioning within the septum to reduce the risk of septal perforator branch injury;
- Patients with interventricular septal hematoma may experience chest pain or dyspnea immediately after transseptal perforation;
- Electrocardiographic abnormalities such as ventricular ectopic beats or NSVT of different morphologies can serve as markers of septal injury;
- Bedside echocardiograms are crucial for early diagnosis and should be repeated the day after the procedure, especially when ventricular arrhythmias are present;
- Troponin elevation, although commonly observed after LBBAP, may not be useful for discriminating this specific complication.

## 5. Conclusions

Interventricular septal hematoma is a rare and recently recognized complication of LBBAP, not previously documented in conventional cardiac pacing techniques. An interesting observation is that ventricular arrhythmias can serve as clinical indicators of this complication. In most cases, computed tomography angiography or coronary angiography is not typically required, while echocardiography plays a crucial role in both diagnosing the hematoma and monitoring its regression during follow-up. Although the clinical presentation of this rare complication can be worrisome, a conservative management approach can be considered appropriate, as complete resolution is usually observed within one month.

## Data Availability

The data underlying this article will be shared upon reasonable request to the corresponding author.

## Abbreviations

ECG	electrocardiogram
LBBAP	left bundle branch area pacing
IVS	interventricular septum
VA	ventricular arrhythmias
VEB	ventricular ectopic beats
NSVT	non-sustained ventricular tachycardia
RV	right ventricle
RBBB	right bundle branch block
HBP	His bundle pacing
